# Improving the Implementation of Patient-Reported Outcome Measure in Clinical Practice: Tackling Current Challenges With Innovative Digital Communication Technologies

**DOI:** 10.2196/60777

**Published:** 2025-02-05

**Authors:** Kelly M de Ligt, Saar Hommes, Ruben D Vromans, Eva Boomstra, Lonneke V van de Poll, Emiel J Krahmer

**Affiliations:** 1 Department of Psychosocial Research and Epidemiology Netherlands Cancer Institute Amsterdam Netherlands; 2 Department of Medical and Clinical Psychology Center of Research on Psychological and Somatic Disorders Tilburg University Tilburg Netherlands; 3 Department Communication and Cognition Tilburg School of Humanities and Digital Sciences Tilburg University Tilburg Netherlands; 4 Department of Research and Development Netherlands Comprehensive Cancer Organisation Utrecht Netherlands

**Keywords:** patient reported outcome measures, quality of life, health communication, delivery of health care, digital sciences, clinical practice: patient reported outcomes, patient reported outcome, digital communication, communication, health management, digital technologies

## Abstract

Implementation of patient-reported outcome measures (PROMs) in clinical practice is challenging. We believe effective communication is key to realizing the clinical benefits of PROMs. Communication processes for PROMs in clinical practice typically involve (1) health care professionals (HCPs) inviting patients to complete PROMs, (2) patients completing PROMs, (3) HCPs and patients interpreting the resulting patient-reported outcomes (PROs), and (4) HCPs and patients using PROs for health management. Yet, communication around PROMs remains underexplored. Importantly, patients differ in their skills, knowledge, preferences, and motivations for completing PROMs, as well as in their ability and willingness to interpret and apply PROs in managing their health. Despite this, current communication practices often fail to account for these differences. This paper highlights the importance of personalized communication to make PROMs accessible to diverse populations. Personalizing communication manually is highly labor-intensive, but several digital technologies can offer a feasible solution to accommodate various patients. Despite their potential, these technologies have not yet been applied to PROMs. We explore how existing principles and tools, such as automatic data-to-text generation (including multimodal outputs like text combined with data visualizations) and conversational agents, can enable personalized communication of PROMs in practice.

## Introduction

Patient-reported outcome measures (PROMs) are instruments that can capture patients’ health status directly through digital administration [1-3]. Patients vary in their skills, knowledge, preferences, and motivations to complete PROMs, as well as their ability to interpret and use the resulting patient-reported outcomes (PROs) for health management. However, PROMs and PROs are not designed to accommodate these differences, often excluding certain populations [4]. This includes older adults, nonnative speakers, individuals with poor health, those lacking social support, people in less privileged socioeconomic positions, or those with low health literacy—defined as “the degree to which individuals can obtain, process, and understand the basic health information and services they need to make appropriate health decisions” [4-6].

Communication is crucial for achieving the clinical benefits of PROMs, for instance when health care professionals (HCPs) discuss PROs with patients. However, the communication processes involved in using PROMs in clinical practice are underexplored [7-9]. We therefore argue that improving communication processes can enhance PROMs implementation. Specifically, personalized communication (information that is customized to the individual) is essential to address the diverse information needs, preferences, and capacities of a broad population [10]. However, personalizing information manually is labor-intensive and costly, which may explain its limited application to PROMs.

To address this, we propose leveraging existing digital technologies to streamline personalized communication for PROMs. Tools such as data-to-text generation, multimodal communication, and conversational agents can offer innovative solutions to improve communication and, in turn, support the broader implementation of PROMs in clinical practice. This paper begins by summarizing communication processes for PROMs in clinical practice and identifying their shortcomings. These shortcomings highlight the limitations of a one-size-fits-all approach, which fails to meet the needs of a diverse patient population. We propose solutions rooted in personalized communication and suggest several digital technologies to support the implementation of these strategies in clinical practice.

## Communication Processes for PROMs in Clinical Practice

**Figure 1:**
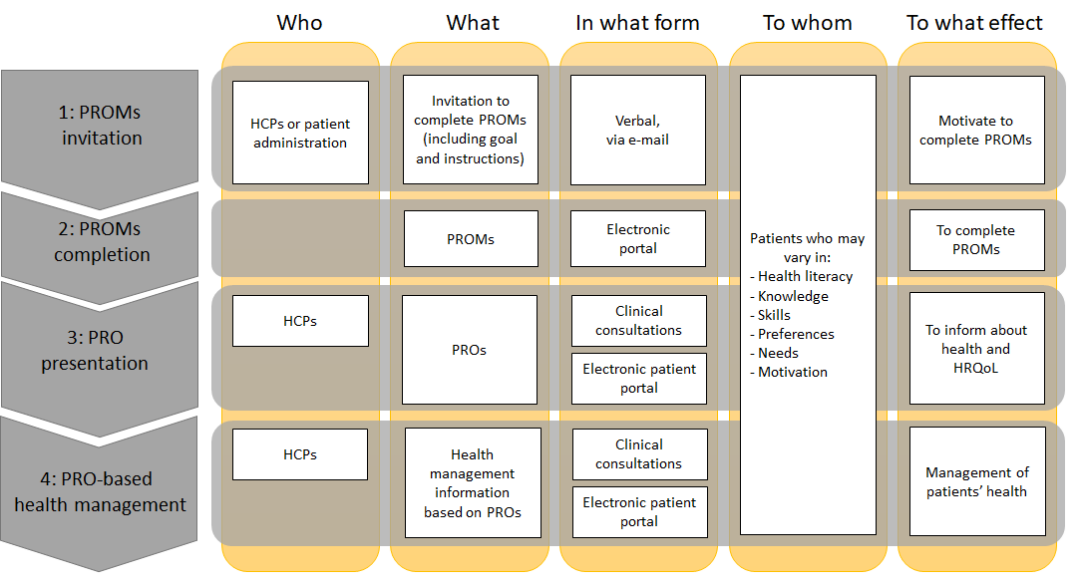
Communication processes for PROMs in clinical practice. HCPs: health care professionals; HRQoL: health-related quality of life; PROMs: patient-reported outcome measures; PROs: patient-reported outcomes.

From many available frameworks, we use the framework of Lasswell [11] to outline key aspects of communication for PROMs in clinical practice: who communicates what, in what form, to whom, and to what effect (Figure 1). We distinguish 4 communication processes, that are PROMs invitation, PROMs completion, PRO presentation, and PRO-based health management.

First, HCPs or administrative staff (“who”) invite patients (“to whom”) verbally or by email (“in what form”), including the goal and practical guidance on how to complete PROMs (“what”) [11,12]. This aims to engage patients to complete PROMs (“to what effect”).

Second, PROMs (“what”) are administered through online portals (“in what form”) for patients (“to whom”) to complete (“to what effect”).

Third, HCPs (“who”) should consistently discuss PROs (“what”) with patients (“to whom”) during consultations (“in what form”) [12,13], providing insights into their health and health-related quality of life (HRQoL; “to what effect”) [12]. Alternatively, patients can occasionally access their PRO reports directly via electronic portals (“in what form”), bypassing HCPs.

Fourth, HCPs (“who”) can translate the PROs into actionable health management information (“what”) to aid patients’ (“to whom”) health management (“to what effect”). Similarly, patients may independently access this information (“what”) through electronic portals (“in what form”), bypassing HCPs.

## Shortcomings, Solutions, and Digital Technologies to Improve Communication Processes

The shortcomings of each communication process are detailed below, along with solutions based on personalized communication and digital technologies to support implementation. Table 1 summarizes the identified shortcomings, proposed solutions, and relevant digital technologies.

**Table 1:** Improving communication processes around PROMs^a^: identified shortcomings, proposed solutions, and relevant digital technologies.

Identified shortcomings		Proposed solutions	Digital technologies to support implementation
**1: PROMs invitation: tailoring invitations by data-to-text to accommodate more patients**			
	Not all patients understand the purpose of completing PROMs, when PROs^b^ will be discussed, and how they benefit, resulting in varying levels of motivation to complete PROMs.	Written invitations could be tailored to accommodate different reading and health literacy levels to better serve patients with varying literacy and health literacy levels.	“Data-to-text” can tailor PROMs invitations to better serve patients with varying literacy and health literacy levels in an automated, data-driven way.
**2: PROMs completion: multimodal communication principles to support PROMs completion**			
	Not all patients are able to complete PROMs, as they have diverse skills, knowledge, and preferences. Yet, PROMs are presented in a single, standardized format.	Presenting PROMs in other formats other than text could better serve patients with lower (health) literacy.	Multimodal communication, such as a video of an interviewer reading the questions, with written text that changes color in synch with the audiovisual playback.
	Not all patients are able to complete PROMs, as they have diverse skills, knowledge, and preferences. Yet, PROMs are presented in a single, standardized format.	Allowing patients to verbally complete PROMs could better serve patients with lower literacy.	Conversational agents integrated into PROMs administration systems would allow patients to complete PROMs verbally.
**3: PRO presentation: data-to-text to convert numeric PROs into comprehensible texts**			
	Not all patients understand PROs because of the way these are presented, and HCPs^c^ frequently omits to explain PROs to patients.	Presenting PROs in a more patient-friendly way to patients.	Data-to-text applications can convert numeric PROs into written texts or narratives.
**4: PRO-based health management: from PROs to health management by conversational agents**			
	HCPs fail to guide patients to use PROs for health management, or patients perceive health management information as not relevant as their information needs differ between patients.	Both HCPs and patients require guidance on how to use PROs for health management.	Conversational agents can break down information into smaller bits, highlight relevant PROs, and provide self-management advice. By tailoring PROs and self-management actions to individual patients, information becomes more relevant.

^a^PROMs: patient-reported outcome measures.

^b^PROs: patient-reported outcomes.

^c^HCPs: health care professionals.

### PROMs Invitation: Tailoring Invitations by Data-To-Text to Accommodate More Patients

Not all patients understand the purpose of completing PROMs when PROs will be discussed, and how they will benefit, leading to varying levels of motivation to complete PROMs. Some patients may perceive PROMs as tools for research or to train HCPs [12-15]. Written invitations could be tailored to different reading and health literacy levels to better serve patients with varying literacy and health literacy levels, ensuring information about the goal and use of PROMs are clearly communicated, potentially improving completion rates.

“Data-to-text” is a digital technology with the potential for tailoring PROMs invitations to diverse audiences in a data-driven manner. A form of natural language generation (NLG), it uses insights from computational linguistics and artificial intelligence (AI) [16]. This technology automates the conversion of data into coherent natural language, typically achieved through a series of computational steps [17]. A standard data-to-text pipeline processes a patient’s data, using a sequence of algorithms to determine the content and structure of the output text. By incorporating self-reported or clinically obtained information about patients' education, literacy, and health literacy levels, the system could generate texts at various readability levels to accommodate a range of reading proficiencies [18].

### PROMs Completion: Multimodal Communication Principles to Support PROMs Completion

Patients have diverse skills, knowledge, and preferences for completing PROMs [4] (Table 1). While digital PROMs can be offered through multiple completion methods [19,20], and flexible administration is recommended [21], often only a single method is provided. As a result, not all patients can complete PROMs. Those facing barriers are particularly older, non-White, lower-educated patients, and those with low health literacy [4,22]. Reducing these barriers could improve completion rates in these groups.

Data-to-text could tailor PROMs to patients’ reading levels, but textual PROMs may still be too lengthy, information-dense, or intimidating for some patients. According to dual coding theory [23] and the cognitive theory of multimedia learning [24], multimodal communication (especially combining text with visuals) may help alleviate these issues [18]. These theories suggest that our working memory processes different types of information through separate channels, each with limited capacity. Using multiple channels, such as visual and verbal information, enhances information transfer. For example, integrating images with text can significantly improve comprehension, as long as the visuals and text are presented together and complement each other [24-27].

Examples of multimodal PROMs already exist [20,28-31], such as the multimedia program of Thumboo et al [28], which delivers PROMs via a touchscreen device. Each question is paired with visual and auditory stimuli – a video of an interviewer reading the question, with accompanying text that changes color in sync with the audio playback. The playback speed can be adjusted to accommodate patients with different reading levels. This approach could be extended to PROM invitations, questions, and PRO-related texts, improving accessibility and comprehension.

Furthermore, conversational agents, which emulate human conversation through text or speech [32], can be integrated into PROMs administration systems, allowing patients to complete PROMs verbally using familiar devices such as computers, smartphones, or tablets [31]. We believe this approach could be especially effective when combined with multimodal or interactive voice response systems that read questions aloud [20,31,33], making PROMs completion more accessible, especially for less literate patients. In addition, conversational agents can overcome language barriers by supporting multiple languages, promoting inclusivity for nonnative speakers [34]. Examples of this have been demonstrated by Mlakar et al [30] and Fenza et al [35], though formal evaluations of their relevance and efficiency are still needed.

### PRO Presentation: Data-To-Text to Convert Numeric PROs Into Comprehensible Texts

Although patients want to reflect on their health and HRQoL before discussions with their HCP [13,14], many are unable to do so. That is, digital PROMs typically grant HCPs but not patients’ access to PROs [7]. Despite this, HCPs often neglect to discuss PROs [8], and patients are generally hesitant to initiate these conversations [13]. When patient do have access to their PROs, these are typically presented in numerical or graphical formats. While patients have expressed a need for personalized numerical information [36,37] and can interpret basic line or bar graphs [19], such formats often lack context and evaluability (ie, how good or bad the scores are) [38], leading to misinterpretation [19,39]. PRO comprehension is influenced by patient’s health literacy [40] and statistical literacy or numeracy skills [41]. However, despite patients’ varying levels for both, PROs are typically presented in a single, fixed format [19]. We believe that improving how PROs are presented will enhance their relevance and value for patients, providing better and more equitable access to the potential benefits of PROs in patient care.

Data-to-text applications could convert numeric PRO scores into written texts, combining numerical information with verbal descriptors to enhance understanding and improve information transfer [42], as studies suggest [12,18,19,43]. We believe both patients and HCPs could benefit from this approach. When discussing individualized outcome data, it is recommended to combine numerical or visual information with verbal descriptors to better align with patients’ needs and capacities [44]. While some patients and HCPs prefer verbal descriptors over “cold” numbers or graphs for discussing outcomes and HRQoL data [36,45], relying solely on words can lead to varied and inaccurate perceptions. Despite its potential, the practical use of NLG for converting PROs into coherent texts is not yet common in clinical settings. The Dutch “Data2Text” project represents an initial exploration into this promising field [46].

Patient narratives, or stories about other patients’ experiences, can help patients understand the meaning of their own PROs [37,42]. When patients recognize PROMs as reflections of their health experiences, HCPs can engage in discussions about how PROs can enhance HRQoL and care [14]. As a form of data-to-text, personalized narratives could be generated based on patients’ PRO scores. This approach was explored in previous research by our group, where it was found to be feasible for clinical practice. Importantly, we discovered that narratives can provide emotional support, complementing numeric data to facilitate discussions about HRQoL during clinical consultations [37]. With further development, a decision algorithm could select relevant stories from a collection of patient narratives, using a patient’s PROs as input.

### PROs Presentation—Based Health Management: From PROs to Health Management by Conversational Agents

In one-third of studies reviewed by Anatchkova et al [3], HCPs failed to guide patients on PRO-based health management. In addition, patients typically use health information in decision-making only when the information is relevant to them, and information needs vary between patients (Table 1) [42]. Improving patients’ access to personalized health information could potentially enhance their care and support better health-related outcomes.

Both patients and many HCPs need guidance on how PROs contribute to care, and how to interpret, discuss, and act on PROs [8,13,14]. Conversational agents can break down information into smaller, manageable pieces, acting as intermediaries by highlighting relevant PROs and providing self-management advice [42,47]. Examples include conversational agents developed for asthma self-management [48] and to promote healthy eating behaviors [49]. For patient dashboards, cognitive load can be reduced by highlighting key information and enhancing evaluability [42]. When PROs are presented numerically or graphically, emphasizing the clinical significance of scores aids interpretation. This can be achieved through color-coding (eg, red for alarming and green for non-alarming scores), using exclamation marks or red circles for critical scores, and adding threshold lines to indicate scores relative to clinical thresholds [19]. We believe this approach could facilitate PRO interpretation and clinical decision-making, accelerating the use of PROs in health management. In addition, for patients, simply receiving health information is not enough — it must be interesting and relevant to them, which varies between patients [42]. Based on patients’ predefined goals and preferences, conversational agents can tailor PRO scores and self-management actions to meet their specific information needs.

## Implementation of Technology-Assisted Communication Processes Around PROMs

With increasing pressure on health care, digital strategies for patient self-management are expected to play a larger role [50,51]. Paradoxically, those who would benefit the most from enhanced self-management support (such as vulnerable populations) are often the ones facing barriers to participation. For instance, lower health literacy is associated with worse health outcomes and healthcare service utilization [52], while lower digital health literacy correlates with lower overall survival rates in cancer patients [53]. While PROMs have the potential to improve symptom management, HRQoL, patient-HCP communication, and patient satisfaction [7,12,13,54,55], vulnerable patients may currently lack access to these benefits. By implementing personalized communication strategies and leveraging digital technologies, we believe PROMs can become more accessible and easier to complete, thereby increasing completion rates. Furthermore, making PROs easier to interpret can improve their usability and value for patients, ensuring that PROMs can more effectively support patient care, particularly for those currently left out.

Despite promising advances, the use of the digital applications described here is not without risks. Data-to-text algorithms, particularly those based on data-driven approaches, present certain challenges. Traditionally, data-to-text systems relied on hand-crafted rules, which were difficult to scale. However, recent advances in machine learning, driven by increased computing power, have streamlined this process, enabling scalability and efficiency [18,56]. Large language models, such as exemplified by ChatGPT, are arguably the culmination of this approach. While these models generate fluent text, they are not always accurate and may contain harmful biases. Large language models rely on massive volumes of human-authored texts, typically sourced from the internet, raising concerns about the exclusion of underrepresented populations (eg, those with a small presence on the internet) and the inclusion of biased or toxic language [57,58]. Therefore, rule-based NLG might be preferable for converting PROs into text, offering more control and reliability. Similarly, while advances in machine learning have enhanced conversational agents with increased dialogue complexity, they introduce risks in natural language understanding, response generation, and patient interpretation, necessitating rigorous monitoring and validation [59]. The safety of conversational agents for patients remains inadequately evaluated [32]. We emphasize that these digital applications require further exploration and testing, despite their clear promise.

In addition to personalized communication, patient-centered communication could improve the use of PROMs in practice [21]. Currently, patients face issues with wording, length of questionnaires, and formatting of PROMs, which can hinder their understanding of questions and answer choices [15,34,39]. Over half of the commonly used cancer-related PROMs to not follow best-practices for plain language use [60]. To improve completion rates, existing PROMs should be revised to use simple, concrete language, avoiding jargon and negations, and eliminate redundant or distracting information [15,61]. Instructions should also be simplified [61-63] and structured with clear orientation statements (eg, “First I will explain the goals of PROMs, then I will show you what the questions are about”) to aid understanding [61,62]. Resources like the “health literacy universal precautions toolkit” can help assess readability and convert medical terms into plain language [63].

To reduce patient burden, PROM should be relevant for individuals [21]. Computer adaptive testing can ensure follow-up questions are tailored to each patient, rather than asking the same set for everyone. The formatting of PROMs should prioritize clarity, with large fonts, clear headings, and ample white space [15,61]. Patient-preferred modifications include removing or altering items, using visuals, simplifying language, and adjusting layouts for better comprehension [29]. Using user-centered design can help meet the diverse needs of patients, considering factors such as clinical conditions, culture, languages, and literacy levels [4]. This approach should also explicitly involve individuals with low literacy skills or learning disabilities, as they are often excluded from PROMs development [64]. By doing so, we can ensure that PROMs are accessible to a wide range of patients, including those with disabilities [20].

Training HCPs about the goals, tasks, and responsibilities related to PROMs is essential for motivating their engagement with PROMs [65,66]. In general, HCPs should discuss or, at a minimum, briefly mention PROs with patients, even when no new clinical information arises from the data [13]. HCP engagement plays a significant role in patients’ motivation to complete PROMs [67]; failing to follow up on PROMs may negatively affect patients’ HRQoL [68]. Further research is needed to assist HCPs in interpreting PROs and converting them into actionable steps for health management [3,13]. That said, some patients may avoid engaging with detailed PRO data [36], highlighting the need for tailoring information to meet patients’ individual preferences for how they receive and process information. Although digital technologies can support communication around PROMs in clinical practice, they may not be able, now or ever, to replace the HCP in addressing patients’ HRQoL.

## Future Implications and Next Steps

Implementing the proposed technologies in clinical practice might present some practical hurdles. For instance, many of the identified applications have not been seamlessly integrated into electronic health records (EHRs) [47,69]. Future health care systems will require EHRs that can safely incorporate digital applications. However, models could be trained without sharing data from EHRs, using techniques like federated learning [70]. Furthermore, trust, which is crucial for patient adherence [71], is challenged by the perceived “black box” nature of AI models. Therefore, it is essential to have human oversight of AI outputs and to maintain transparency about data sources and methods [59]. Both HCPs and patients should become more AI-literate, and have a basic understanding of the capabilities and limitations of AI models [70]. To ensure that readers can assess the credibility of generated texts, it is essential to explain that texts come from a computer model, and not a fellow human [57].

The successful implementation of PROMs in clinical practice involves complex interactions between care processes, technological development, and human behavior [66]. Often, PROMs implementation is led by HCPs or researchers, potentially resulting in a lack of recognition of these complex interactions. This paper was written by a multidisciplinary team with expertise in PROMs, implementation science, health communication, and digital technologies. We have provided a comprehensive overview of key communication processes around PROMs. Future work could deepen this assessment by studying the effects of context and noise in communication, which are aspects currently missing from the application of the Lasswell model [11]. With ongoing advancements in AI, which may help mitigate the risks associated with the proposed digital applications, we envision a future where personalized communication strategies and digital tools can overcome current implementation challenges, ensuring that PROMs are applied inclusively to benefit all patients.

## Abbreviations

AI: artificial intelligence
EHR: electronic health record
HCP: health care professional
HRQoL: health-related quality of life
NLG: natural language generation
PRO: patient-reported outcome
PROM: patient-reported outcome measure

## Data Availability

Data sharing is not applicable to this article as no data sets were generated or analyzed during this study.
